# DO STEPCHILDREN PROVIDE DEMENTIA CARE TO AGING BIOLOGICAL PARENTS COMMENSURATE WITH THEIR LEVEL OF NEED?

**DOI:** 10.1093/geroni/igae098.1319

**Published:** 2024-12-31

**Authors:** Merril Silverstein, Seonhwa Lee, Martin Lakomy

**Affiliations:** Syracuse University, Syracuse, New York, United States; Syracuse University, Syracuse, New York, United States; Mendel University, Moravskoslezsky kraj, Czech Republic

## Abstract

The research in this presentation addresses the critical issue of whether divorce, remarriage/repartnering, and stepfamily formation have weakened adult intergenerational bonds due to stepchildren’s weaker commitment to the care of their vulnerable stepparents. A life-course approach is taken to link early relationship characteristics to later care trajectories. We use data from the Longitudinal Study of Generations to examine whether earlier kinship strength with stepmothers and stepfathers increases the amount of care stepchildren provide to meet the needs of their aging stepparents between1997-2022. We use latent growth curve analysis to examine whether the rate of increase in care to stepparents with advancing dementia and functional impairment is stronger when stepchildren in earlier adulthood evaluated their relationship with stepparents as more “kin-like” and “family-like.” In comparison, we also examine rate of change in care for biological mothers and fathers by their levels of functional impairment. We also consider structural characteristics of step-relationships, including the child’s age when the relationship was formed and the number of years the child lived with the stepparent. We expect that earlier reported kinship strength with stepparents will moderate the care response to stepparents’ impairment such that it will approach the care response to biological parents. We expect the severely impaired stepmothers to elicit more care from stepchildren when compared to severely impaired stepfathers. Analyses reveal the conditions under which stepchildren serve as a vital resource to their vulnerable older stepmothers and stepfathers with dementia in complex aging families, which have become increasingly prevalent but remain under-investigated.

